# Graded expression of source memory revealed by analysis of gaze direction

**DOI:** 10.1371/journal.pone.0188727

**Published:** 2017-11-27

**Authors:** Andrew Talk, Inés Antón-Méndez, Bronte Pennefather

**Affiliations:** 1 Discipline of Psychology, School of Behavioural, Cognitive, and Social Sciences, University of New England, Armidale, New South Wales, Australia; 2 Discipline of Linguistics, School of Behavioural, Cognitive, and Social Sciences, University of New England, Armidale, New South Wales, Australia; University of Akron, UNITED STATES

## Abstract

During source memory studies, knowledge of some detail about the context of a previously experienced item or event is tested. Here, participants attended to different objects presented at different quadrants on a screen. In a later test phase, a single object was presented in all four quadrants, and participants verbally reported whether the object was new or previously seen (item recognition), and if it was previously seen, they indicated the original screen location (source memory). We combined this test with eye-tracking to determine whether attention to an object during encoding would correlate with later recognition of the object and memory of its source location, and whether eye movements at test can reveal attention to the correct source location in the absence of correct explicit verbal responses. The amount of time spent looking at an object during encoding was not related to later object recognition or source recollection. However, we found that eye movements at test reveal retention of source information about an object in the absence of accurate retrieval of source information as assessed by verbal response. When participants correctly recognized an object but incorrectly indicated the source information, significantly more time was spent looking at the correct source location than to incorrect, non-selected locations. Moreover, when participants correctly recognized an object but said they could not remember the source information, significantly more time was spent looking at the correct source location. These results are consistent with the hypothesis that eye movements are sensitive to attention or other graded mental processes which can underlie the retrieval of source memories that can then be expressed verbally in a thresholded manner.

## Introduction

As humans go through daily life, memory for the context or location in which objects or events were experienced can be important. For example, it can be important for us to remember where we last saw objects such as our car keys so we can retrieve them when necessary. Or, it can be important for us to remember the contexts in which we previously experienced people we meet so we can respond appropriately to them. This type of memory is commonly referred to as source memory, and is often contrasted with recognition of the object itself [1]. The objective of this study was to examine attentional correlates of source memory encoding and retrieval using eye tracking technology.

Source memory tests are considered by some investigators to assess the same construct underlying tests of episodic memory (e.g. [2]). Episodic memory is often assessed using participant self-reports of the subjective experience of remembering as recollection versus familiarity. For example participants may be asked whether they can replay the prior episode in their minds or whether they know the information without episodic recall [3–5]. It is also possible to use reduced or simplified experimental procedures to test encoding and retrieval of information without a verbal report of subject experience [6]. These procedures are more suitable for studies where it is not feasible to obtain a verbal report, such as during functional imaging [7], during tests of episodic memory in young children [8], or during tests of episodic-like memory in animals [9]. Source memory tests are one class of reduced procedures, in which both recognition of a previously experienced object or event and identification of an aspect of the original context can be assessed [7, 10–13].

Source memory depends on binding and retention of all the features in the experienced context during an encoding episode. Then at test, memory of one or more contextual features is assessed along with recognition of a specific object that occurred during encoding. In one common source memory procedure [7, 10–13] participants are asked to attend to images of objects that are incidentally presented at different quadrants on a screen. Then in a later test phase, the images are presented at the screen center and participants are asked to respond whether the object is new or previously seen (item recognition), and if it was previously seen, to indicate the original screen location (source memory test). In other studies, subjects have been asked to indicate in what color font recognized words were originally presented [14], whether recognized sentences were originally presented in a male or female voice [15], or where remembered trivia items were originally learned [16]. In such studies, correct answers on the source memory component of the test could then be based on either recollection of the prior episode or familiarity with and recognition of the correct source possibility.

Dual-process models have been proposed in which familiarity and recollection are main cognitive processes involved in remembering [1, 17]. According to these models, familiarity involves a fast and automatic process that allows for recognition of a previous experience without retrieval of contextual details from the encoding experience, while recollection is a slower process that involves retrieval of details about the surrounding context of the encoding episode. A wide range of experimental evidence supports models for a dual-process memory systems (for review, see [17]). Recollection has been considered as an essential component for source memory [18], but models of familiarity-based recognition can also account for source memory, and a variety of empirical evidence has suggested that familiarity contributes to source recognition in studies in which the test of source requires participants to recognise the correct source from a set of possibilities [1, 17]. This means that measures of source memory that do not rely on verbal reports of subjective recollection cannot be unambiguously interpreted as recall rather than recognition of, or familiarity with, the source possibilities.

Models of the neural activity associated with memory encoding and retrieval have suggested that retrieval is associated with activation of the same patterns of neural activity that occurred as a result of sensory experience during the time of encoding. Several studies have supported this type of model by demonstrating that the neural systems that are active during encoding are also active during retrieval [19–21]. Interaction between the stored memory traces and environmental cues present during retrieval is thought to be key, in that the pattern of activity elicited by the environmental cues overlaps the stored memory trace and completion of activation of the stored pattern occurs [22–24]. Moreover, pattern separation must occur with accurate episodic memory retrieval in that multiple patterns of neural reactivation are possible during memory retrieval but one pattern resolves into a distinct representation [25, 26]. Incorrect pattern completion or pattern separation may thus lead to memory failure or to false memories. There has been recent discussion about whether source memory retrieval is a thresholded all-or-nothing process or whether it can be graded [27–29]. It is possible the process is both thresholded and graded. Recent experimental evidence suggests a threshold for retrieval, but also that, when successful, retrieval can have varying levels of precision or accuracy [30].

An increasing number of studies have demonstrated that eye movements can reveal aspects of memory for the spatial positions of elements within observed scenes even in the absence of a verbal report about memory for those elements. For example, Ryan et al. [31] presented participants with pictures containing scene elements with different relationships relative to each other. At a subsequent test, eye movements were monitored as participants viewed scenes that were either novel, repeated, or repeated with one of the elements transposed to a new location. The participants had more fixations to the critical regions of scenes when they contained transposed elements even when they reported being unaware that the picture had changed. Other studies have demonstrated that information about the locations of particular faces in arrays can be expressed through gaze directions [32, 33]. This gaze direction effect has been found to correlate with recall of the original location of faces as assessed through verbal responses, but in some cases can occur even when participants are unable to make correct verbal responses [34].

Here we studied the temporal dynamics of eye movements during a source memory procedure. One goal was to see whether attention to an object during encoding would correlate with later recognition of the object and of its source location. A second goal was to see whether eye movements at test reveal attention to correct source locations as subjects successfully retrieve a source memory, cannot retrieve a source memory, or retrieve a false source memory.

## Method

### Participants

Twenty four healthy participants volunteered to participate in the experiment. The participants were all undergraduate students at the University of New England, Australia. There were 11 female participants and 13 male participants whose ages ranged from 19–24 years (*M* = 20.33). The experiment was approved by the Human Research Ethics Committee of the University of New England.

### Materials

A total of 96 colored images of common objects were employed. The images were developed and described by Cansino [12]. From the pool of images, a set of 64 images were selected to be displayed during the encoding phase. During the retrieval phase, 32 images from the encoding phase were used again, along with 32 new images. The stimuli were presented on the screen of a laptop computer (1366 × 768 pixels; 60 Hz) using e-Prime software version 2.0. A Tobii Technology X2-30 eye tracker was positioned below the screen to track eye movements. The subjects were seated so that their eyes were 50–80 cm from the screen.

### Procedure

The experiment was conducted in a single session that consisted of an encoding phase, a retention interval, and a retrieval test phase. Eye movements were tracked during the encoding phase and the test phase. Before beginning the study, each subject read and signed an informed consent form and filled out a computerized demographic questionnaire. Participants started the experiment by calibrating their eyes to the eye-tracking software.

**Fig 1:**
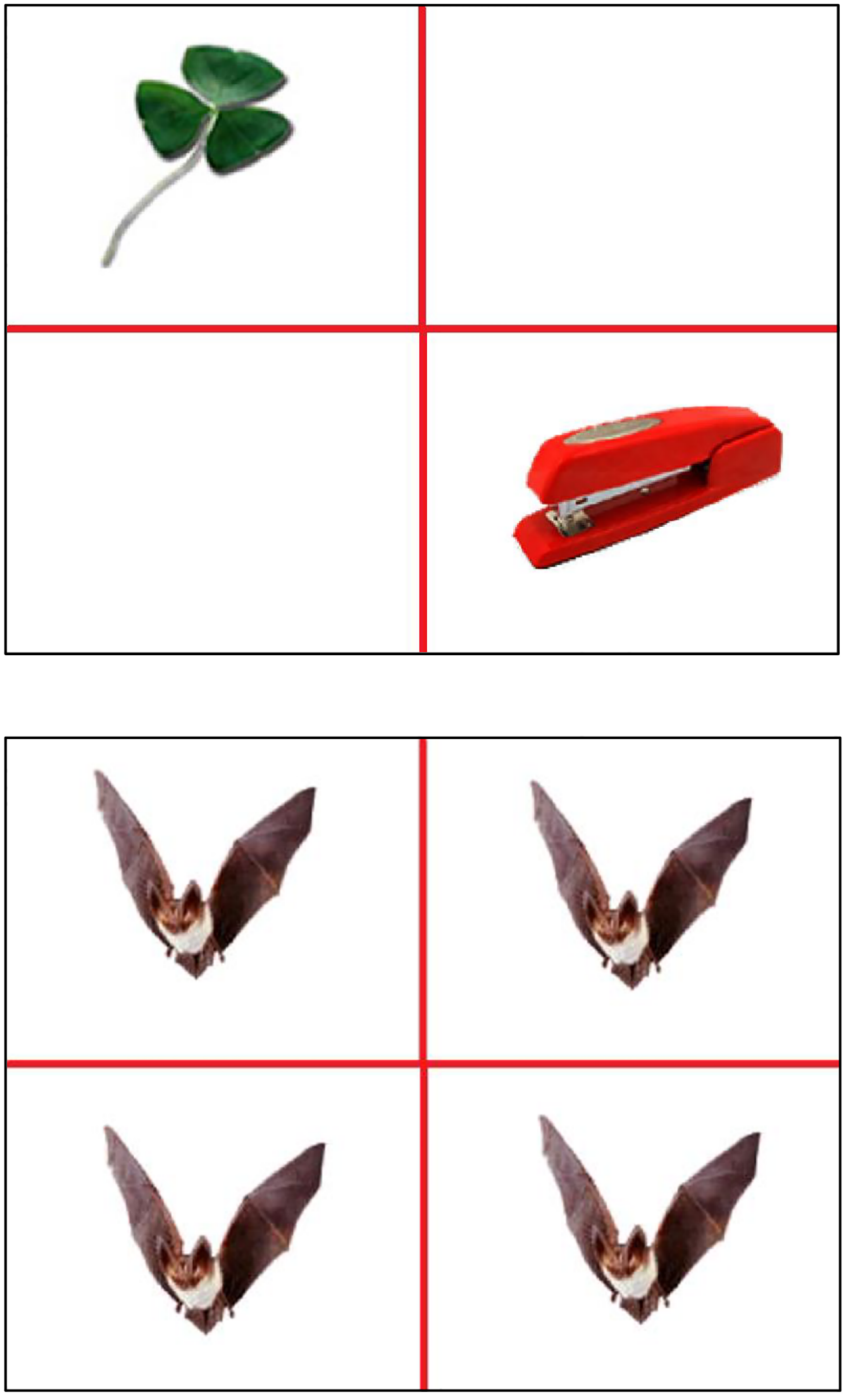
Examples of stimuli layout. In the encoding phase (top panel), a red cross divided the screen into quadrants and the stimuli were presented randomly in opposite quadrants. In the test phase (lower panel), a red cross divided the screen into quadrants and the stimuli were presented in all four quadrants.

Once the calibrations were satisfactory, the encoding phase began, which lasted for approximately 6 minutes. The participants were presented with an illustrative encoding slide and instructed to study the objects that appeared on screen for a later test. After the illustration slide was shown, a series of 32 additional slides was presented. Each encoding slide was divided into four quadrants by a red cross. In two of the quadrants randomly selected pairs of objects were presented. Participants were not instructed to remember image locations or image pairings. Unknown to them, one of the objects from each slide would be part of the retrieval test (the target) and the other would not be seen again (the distractor). Quadrants that contained stimuli were always diagonally opposed: either top left and bottom right, or top right and bottom left (Fig 1, top panel). These screen positions, as well as the position of the object that would be presented again later, were counterbalanced across the session and presented in individually randomized orders. Before each new slide was displayed, the participants were presented with a fixation cross for 1000 ms to center their focus. Each slide was presented for 3000 ms, after which the images and the quadrants disappeared and were replaced with the message “press space to continue”. By pressing spacebar, the participant could then choose when they would see the next slide. On average, participants took 1439 ms to continue onto the next slide.

Immediately after the encoding phase there was a ten minute retention interval. During this interval participants completed a distractor task that involved solving simple arithmetic problems. The participants were asked to select the correct count-down series by threes out of 3 options. They had to do this for 10 different numbers ranging from 17–32.

For the test phase, participants were presented with test screens divided into four quadrants by a red cross. The same object was displayed in each quadrant (Fig 1, bottom panel). The subjects were first presented with a sequence of three illustrative test trials and advised that after viewing the objects in each trial they would be asked to either point with their hand to the quadrant that they had previously seen the image in, say “new” if they had not seen the image previously, or say “don’t know where” if they had seen the image before but could not recall where its previous location was. For each test trial, a fixation cross in the center of the screen appeared for 1000 ms before each slide was presented to enable the participant to center their focus. The objects appeared for 3000 ms, then they were removed while the red dividing cross remained. At that point, the verbal responses from the subject were collected. These verbal reports were recorded by the experimenter. The subjects were presented with 64 test slides, 32 of which contained objects that were previously shown in the encoding phase and the other 32 contained new objects. The sequence of new images and previously presented images in the test screens was individually randomized.

### Coding of responses

The third author noted participants’ response as they were being given and later coded these responses according to the following guidelines:

- Incorrect item recognition—previously seen objects which were labelled as new by the participant.
- Correct item recognition—previously seen objects which were not labelled as new by the participant.
- Correct source location—previously seen objects which were recognized and for which the participant had pointed to the correct quadrant.
- Incorrect source location—previously seen objects which were recognized but for which the participant had pointed to an incorrect quadrant.
- Don’t know where—previously seen objects which were recognized but of which the participant said they did not know the location.

New objects could also be correctly identified as new or falsely considered to have been seen before. These were not analyzed further.

### Data analysis

The eye tracker sampled gaze direction at 30 Hz, or about once every 33.33 ms. The data were then coded as gaze directed within the target, in one of the other filled quadrants, or as no gaze detection, which was assumed to reflect gaze outside the areas of interest. For data collected during encoding, the averaged amount of time (expressed as number of samples in which the participant’s gaze was detected) looking at the target or distractor quadrant across the entire trial was calculated. For data collected during the test, the time looking at the target or one of the competitor quadrants was calculated for each 100 ms time bin.

Two types of analyses were carried out. To see the effect that initial time spent looking at an object during encoding had on the likelihood that the object was later recognized and the source correctly indicated, the number of samples in which gaze was detected as falling on a given quadrant during a trial’s encoding phase was modelled with linear mixed-effects logistic regression [35] using the lme4 package in R version 3.1.3 [36, 37], and lmerTest to calculate the resulting coefficients’ degrees of freedom (Satterthwaite approximation) and associated p-values [38]. The models included the fixed factors (predictors) of quadrant content (target or distractor) and response accuracy (correct or incorrect item recognition, or source memory). A maximal random effect structure including random intercepts and slopes over subjects and items was always attempted first [39]. However, non-convergence issues in the case of the model analyzing source memory forced a simplification of the random effects which was implemented by eliminating the higher order factors [39].

In contrast with the encoding phase, where we were only interested in whether increased attention to the target object would result in increased memory accuracy, in the test phase it was important to consider eye movement patterns across time. The total number of samples in which gaze was detected in a quadrant could mask a different temporal pattern (e.g., the target may be preferentially fixated first and, once identified, participants could turn their attention to other quadrants). To this end, growth curve analyses (GCA; [40]) were carried out on the number of samples in which gaze was detected as falling on a given quadrant within 100 ms time bins starting from the time the test slide came into view. The analysis contrasted the number of samples in which gaze was detected as falling on at different quadrants during the test phase as a function of whether the quadrant had originally contained the target or was one of the competitor quadrants, and whether it was the quadrant selected by the participant for their verbal response (predictors).

The overall time course of such gaze-detections was modelled with a first (linear), a second (quadratic), and a third (cubic) orthogonal polynomial; and always a maximal random effect structure consisting of random effects of time terms over subjects and over subject-by-quadrant content. In these analyses, a significant intercept for a given main effect or interaction indicates an overall difference of total gaze-detections associated with different quadrants due to that main effect or interaction—i.e., whether there were differences in the total number of gaze-detections for different quadrants regardless of how these gaze-detections were distributed across time. An effect of the linear term gives an indication of whether the number of gaze-detections rose or fell differently according to the main effect or interaction—that is, whether there were differences in the slopes associated with looks to different quadrants. Finally, an effect of the quadratic or cubic terms indicates differences in the shape of the curve across the time window suggesting more complex differences in the dynamics of eye movements associated with different quadrants as a result of the main effect or interaction.

## Results

### Task performance

The percent of previously seen objects that were correctly recognized was 83.2 ± 2.6 (mean ± s.e.), leaving 16.8 ± 2.6 of previously seen objects not recognized. Of previously seen objects correctly recognized, 66.4 ± 2.8 locations were accurately indicated, 22.1 ± 3.0 locations were incorrectly indicated, and 11.4 ± 2.7 locations could not be remembered. The percent of new objects correctly identified as new was 86.3 ± 2.2.

When participants indicated an incorrect source location, they were more likely to choose a quadrant adjacent to the correct quadrant than choose the opposite quadrant. In trials where there was an incorrect source location, the adjacent quadrants in the horizontal direction were chosen 33% of the time, adjacent quadrants in the vertical direction were chosen 43%, and the opposite quadrant was chosen 24% of the time. This result suggests that the mistaken quadrant choice was probably not based on an overall recollection of the configuration of objects displayed at the encoding session (i.e. in opposite quadrants).

### Eye movements during the encoding phase

**Fig 2:**
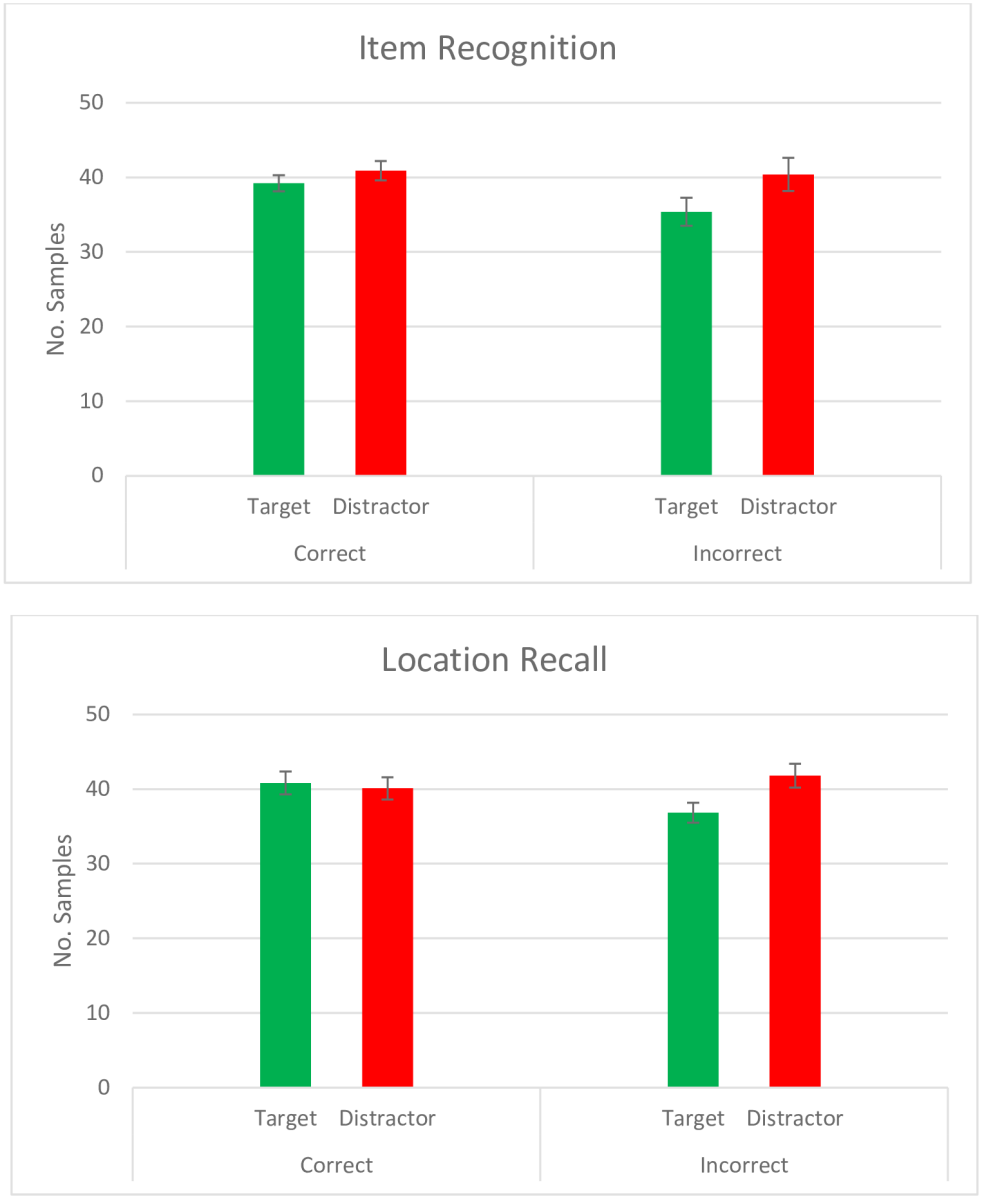
Eye-tracking samples (per trial/per subject) with gaze detected during encoding according to quadrant content. The error bars reflect standard error. Items were divided according to whether the item was later recognized (top panel, number of trials across subjects was 768) and, if recognized, whether its source location was correctly remembered (lower panel, number of trials across subjects was 639).

Fig 2 shows the average number of samples in which gaze was detected as falling on the target and distractor quadrants (i.e., the quadrant containing the object that would be presented during the later test phase and the quadrant that would not) per trial per person during the complete 3 seconds of the encoding phase trials. The top panel shows number of such samples according to whether the object was later correctly recognized, and the bottom panel shows number of such samples according to whether the source location of the object was later correctly remembered.

A comparison of number of samples for which gaze was detected within different quadrants per trial (*N* = 768) according to their content (target vs distractor) and whether the target object was later recognized did not result in statistically reliable differences between targets and distractors overall (*Quadrant content estimate* = -3.27, *SE* = 3.98, *p* = .418), or between trials in which the object was later correctly recognized vs when it was not recognized (*Item recognition estimate* = 0.91, *SE* = 2.39, *p* = .708). The interaction between these two factors was also not significant (*Interaction between quadrant content and item recognition estimate* = 0.97, *SE* = 4.23, *p* = .821).

Analyzing number of samples for which gaze was detected within different quadrants as a function of quadrant content and whether the source had been correctly indicated or not (excluding trials where the object had not been correctly recognized, *N* = 639) again failed to show an effect of quadrant content (*Quadrant content estimate* = -4.02, *SE* = 2.79, *p* = .154), an effect of source memory (*Source memory estimate* = -0.71, *SE* = 1.58, *p* = .657), or an interaction between the two (*Interaction between quadrant content and source memory estimate* = 2.58, *SE* = 1.99, *p* = .194).

### Eye movements during the test phase

**Fig 3:**
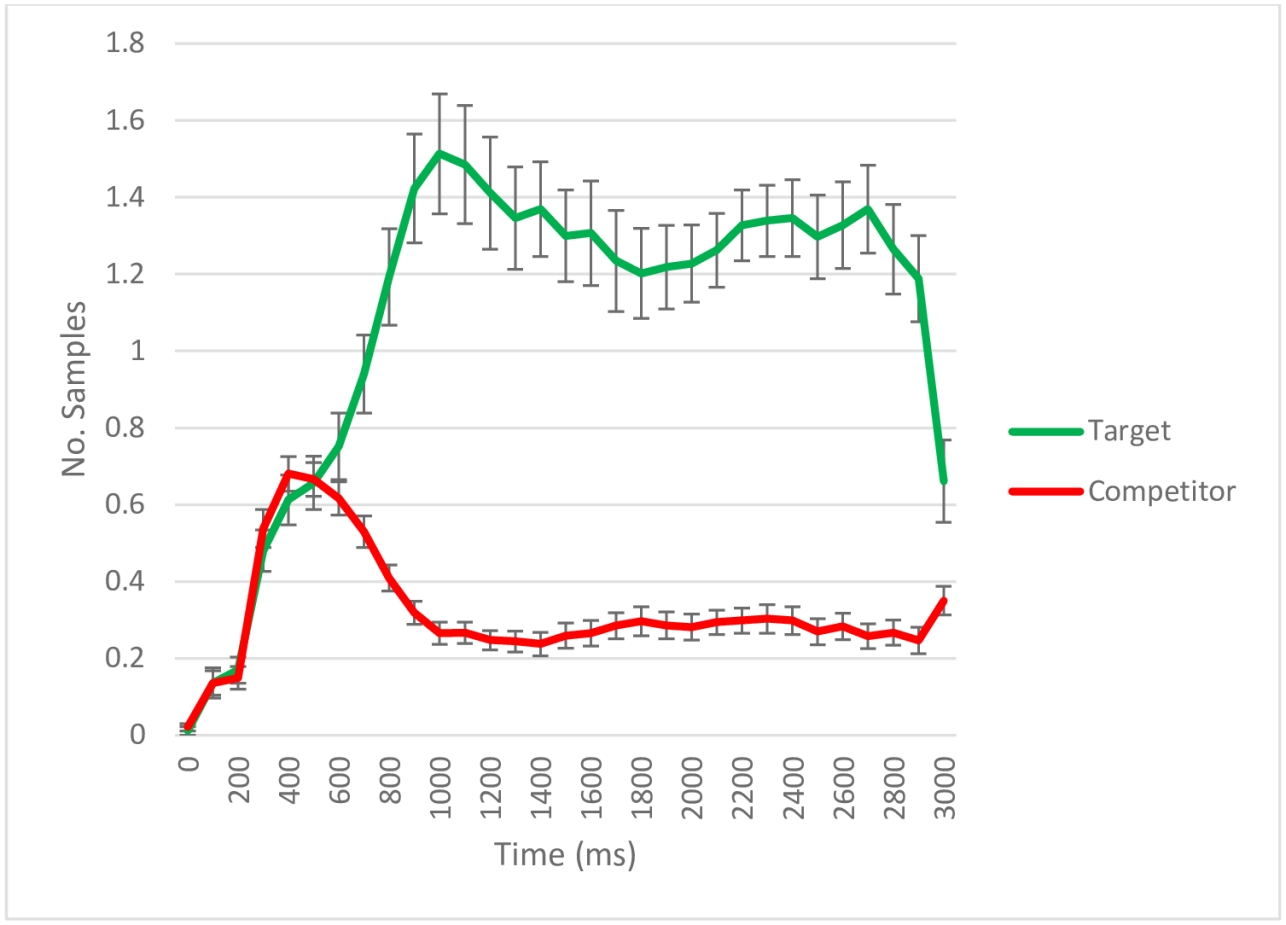
Eye-tracking samples (per trial/per subject) with gaze detected during retrieval according to quadrant content for trials with correct source memory. Data aggregated on 100 ms time bins. The error bars reflect standard error. The total number of trials across subjects was 431.

**Fig 4:**
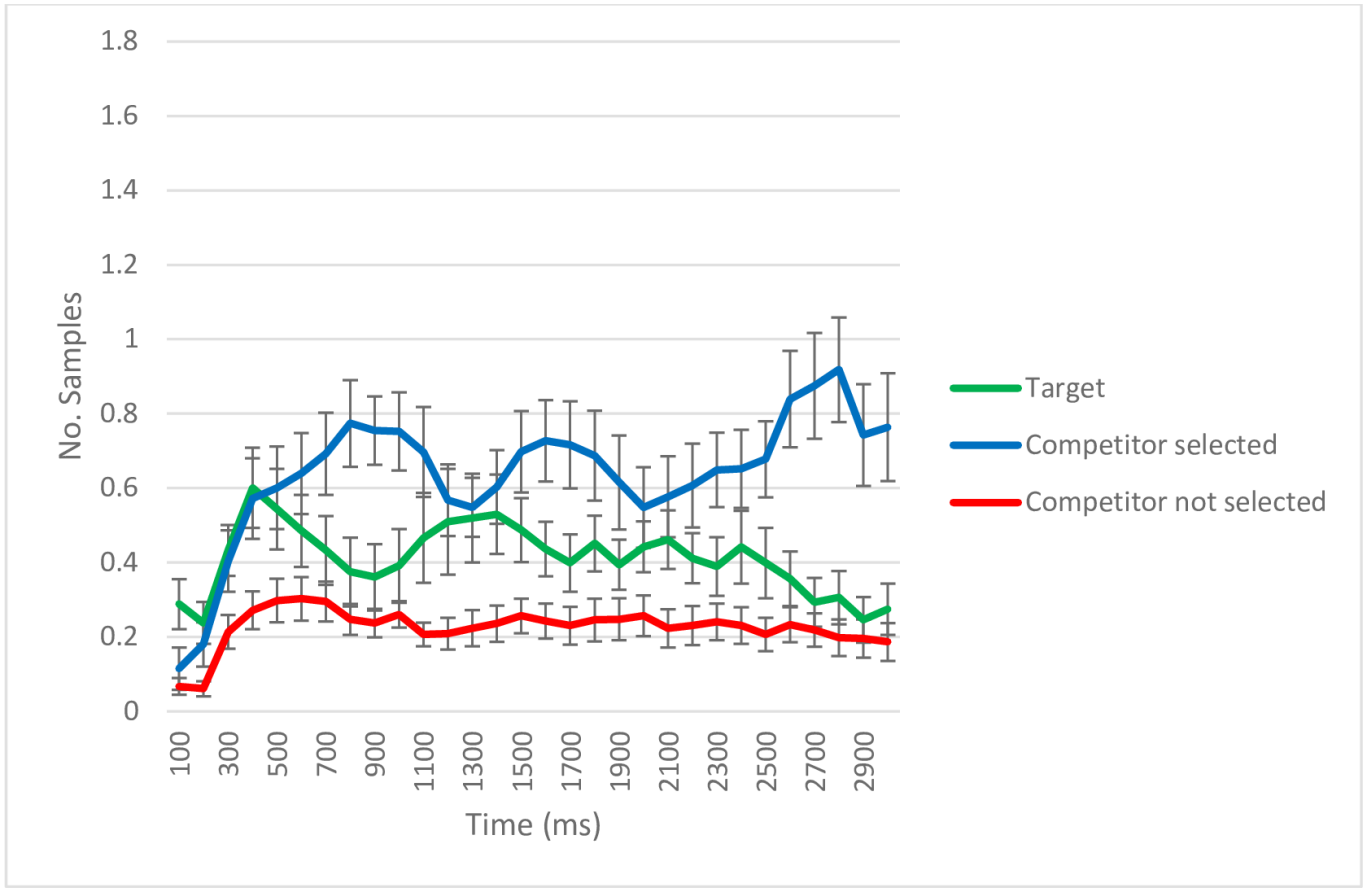
Eye-tracking samples (per trial/per subject) with gaze detected during retrieval according to quadrant content for trials with incorrect source memory. Data aggregated on 100 ms time bins. The error bars reflect standard error. The total number of trials across subjects was 139.

**Fig 5:**
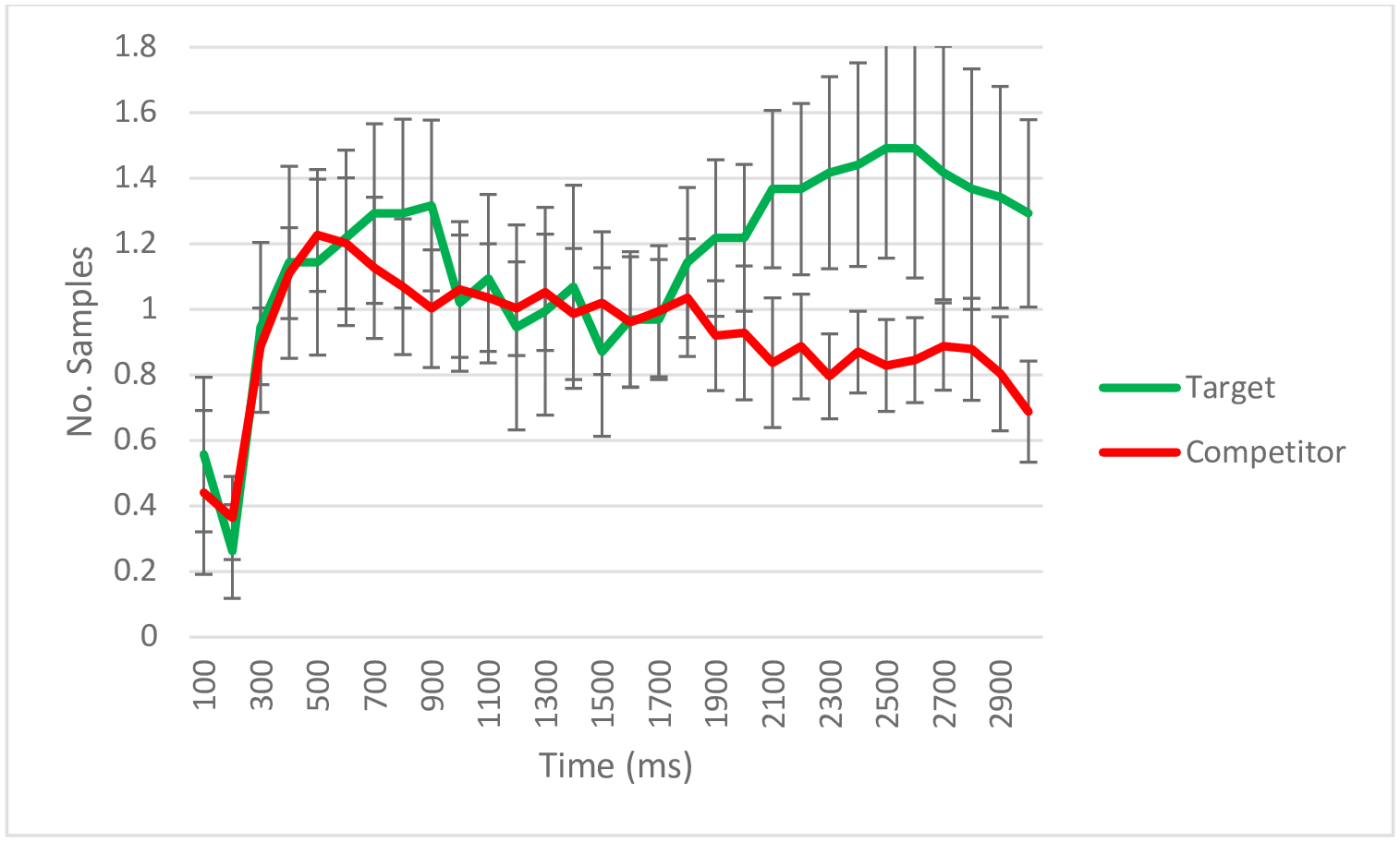
Eye-tracking samples (per trial/per subject) with gaze detected during retrieval according to quadrant content for trials with ‘don’t know where’ responses. Data aggregated on 100 ms time bins. The error bars reflect standard error. The total number of trials across subjects was 69.

**Fig 6:**
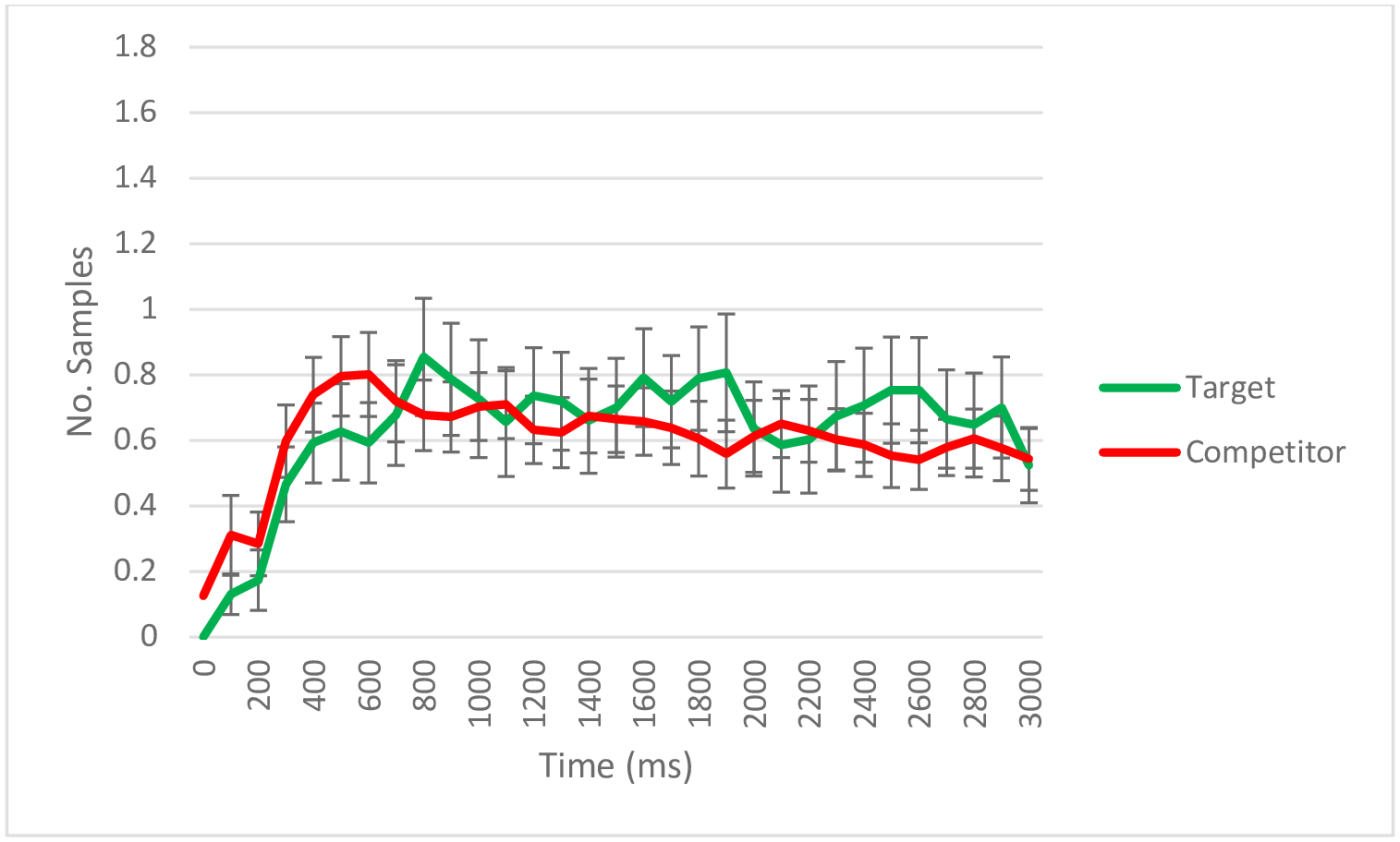
Eye-tracking samples (per trial/per subject) with gaze detected during retrieval according to quadrant content for trials with incorrect item recognition. Data aggregated on 100 ms time bins. The error bars reflect standard error. The total number of trials across subjects was 129.

Figs 3 to 6 show the number of samples in which gaze was detected as falling on the target and competitor quadrants within 100 ms time bins per trial per person in the test phase. Each figure shows the dynamics of the eye movements for different subsets of the trials depending on correct or incorrect verbal responses. There were no a priori predictions regarding the specific time course of eye movements. Therefore, we chose a time window of interest on the basis of the observable patterns for the default case: when participants correctly recognize an object and remember its source location. Visual inspection of eye movement patterns in these conditions (Fig 3) showed a window from 500 ms until the slide disappeared (3000 ms) in which participants preferentially looked at the quadrant containing the target which will be selected. In consequence, we focused our analyses on the interval from 500 ms to 3000 ms.

For trials in which the object source had been correctly indicated (*N* = 431; Fig 3), the GCA showed the number of samples with gaze detections within different quadrants to be dependent on quadrant content (target vs. competitor) both with respect to total number of gaze detections, greater for target quadrants (*Intercept estimate* = 469.04, *SE* = 32.36, *p* < .001), and with respect to the dynamics of eye movements across time (*Linear term estimate* = 487.57, *SE* = 87.26, *p* < .001; *Quadratic term estimate* = -469.72, *SE* = 79.12, *p* < .001; *Cubic term estimate* = 385.42, *SE* = 26.54, *p* < .001). This indicates that the target quadrant that the participant intended to name in their response was looked at more often than the other quadrants on the slide.

We also analyzed the eye movement patterns during the test phase for trials where one of the competitors had been wrongly selected (i.e. false memory trials; *N* = 139; Fig 4). The target was looked at more often than the competitor not selected (*Intercept estimate* = 60.01, *SE* = 19.05, *p* = .002) and less often than the competitor selected (*Intercept estimate* = -88.19, *SE* = 19.04, *p* < .001), which itself was also looked at more often than the other competitors, the ones not selected (*Intercept estimate* = 148.20, *SE* = 19.04, *p* < .001). As for the temporal dynamics of eye movements, the target was significantly different from the competitor not selected on the quadratic term (*Linear term estimate* = -1.36, *SE* = 75.44, *p* = .986; *Quadratic term estimate* = -131.88, *SE* = 59.50, *p* = .027; *Cubic term estimate* = 2.59, *SE* = 23.07, *p* = .910), and from the competitor selected in the cubic term and marginally on the quadratic term (*Linear term estimate* = -40.69, *SE* = 75.44, *p* = .590; *Quadratic term estimate* = -114.02, *SE* = 59.50, *p* = .055; *Cubic term estimate* = -78.53, *SE* = 23.07, *p* < .001). The two types of competitors were significantly different from each other on the cubic term (*Linear term estimate* = 39.33, *SE* = 75.45, *p* = .602; *Quadratic term estimate* = -17.86, *SE* = 59.50, *p* = .764; *Cubic term estimate* = 81.13, *SE* = 23.07, *p* < .001).

We analyzed the eye movement patterns during the test phase for trials where source memory failed and the participant did not select any quadrant after having recognized the object (i.e. the participants responded with “I don’t know” or similar when asked to indicate the source information; N = 69; see Fig 5). The GCA on those trials showed a significant difference on the linear term (*Linear term estimate* = 40.91, *SE* = 14.93, *p* = .006), which stems from a late tendency to predominantly fixate within the correct target quadrant over the incorrect competitor quadrants. This tendency appears starting at 2000 ms after slide onset. No other effects were significant (all *p*’s > .127).

Finally, we analyzed the time course of gaze detections within target and competitor quadrants for those trials in which the previously viewed object was not recognized and was incorrectly declared new (*N* = 129; Fig 6). In this case, no significant differences were found (all *p*’s > .330).

## Discussion

Our objectives in this study were to determine whether attention to an object during encoding would correlate with later recognition of the object and retrieval of its source location, and to determine whether attention to the correct source location at test occurs even when verbal expression of that source location is incorrect or has failed. Consistent with prior studies [7, 10–13], our participants were able to recognize previously seen objects and indicate the screen location where the objects originally occurred. However, the subjects also made errors in failure to indicate the original source locations of objects, in indicating incorrect locations, or in failure to recognize previously seen objects.

Regarding whether visual attention to objects during the encoding session is related to later recognition of those objects and later retrieval of source information about the objects, analysis of gaze direction revealed that response accuracy was not determined by how much an item was initially attended to during the encoding phase. There were no statistically reliable overall differences between how often targets and distractors were looked at during encoding, and no dependency was found of response accuracy on the amount of time spent looking at particular quadrants during encoding. Other studies have demonstrated an effect of attention on source encoding by dividing attention during the encoding session [41] or by using emotionally valenced items [14]. Also, the fact that source memory is sensitive to frontal cortical lesions may be evidence that attention during encoding is important for memory, as frontal cortical dysfunction impairs attention [15, 42]. One possible reason for the discrepancy in the literature is variation across studies in amount of time the participants are afforded to encode the items. In our study the subjects could scan the objects for three seconds, which may have been sufficient to encode both objects. Therefore, during encoding, subjects divided the time similarly between the two objects in each slide. Given all objects were similarly attended to, it is not surprising that attention was not found to be a critical factor in which objects were later remembered.

Regarding the evidence that we collected from eye movements during the source memory test, it clearly shows attention to the original source location on those trials where the subjects provided incorrect explicit location responses or provided a response indicating they didn’t remember the location. During trials in which the object was recognized but an incorrect source location was indicated, the most time was spent looking at the quadrant the subjects would incorrectly select. However, significantly more time was spent looking at the correct source location than at incorrect, non-selected locations. In fact, the distribution of looking across the different quadrants and the dynamics of the eye movements during the retrieval phase suggests competition between the correct location and a wrong alternative (see Fig 4). In these cases, the target quadrant may have attracted eye movements either because it was explicitly considered an alternative to the quadrant eventually chosen or because, despite a decision to report another quadrant, it was recognized as a possible source. We collected further evidence from eye movements which shows attention to the original source location on trials where the participants failed to explicitly indicate any source location. On trials in which the object was recognized but participants responded with “I don’t remember” or similar when asked to point to the original location of the object, significantly more time was spent looking at the correct source location than at incorrect competitor locations starting 2000 ms after slide onset. These two results indicate that accurate source information can be expressed through gaze direction even when verbal expression of that information is incorrect or has failed.

These findings related to eye movements during source memory retrieval are consistent with models suggesting that even though the verbal response associated with retrieval of source memory may be dichotomized as correct or incorrect, underlying processes of source memory are graded. It is possible our subjects were experiencing explicit and conscious partial source recollections and then screening them out in verbal reports or providing incorrect responses depending on their confidence (see [43,44]). Eye gaze cannot then be assumed to reflect memory processes wholly outside awareness in those trials, but only memory processes (whether conscious or not) that the subject withholds from verbal responding. If participants were basing responses on as sense of familiarity with a particular source location then that also would be graded.

Source memory has been defined as the retrieval of contextual details that were incidentally acquired during the prior observation of a remembered item, and is contrasted with item memory which reflects recognition of the item itself [16]. Item recognition and source memory have been widely thought of as separate processes that involve different brain systems [7, 11, 42] and degrade at different rates across ageing [10]. The neural processes underlying recognition of items has been proposed as continuous and underlying weak to strong familiarity, while neural processes underlying source memory have been thought to operate in a threshold manner in which subjects either recall or fail to recall a past event [27]. However, a growing body of behavioral evidence indicates that source memory retrieval can be revealed as a continuous process if participants are allowed to make responses along a graded scale of memory strength [45–48]. In a typical study [49], participants initially heard a male or a female voice saying words, and then during the test phase saw words printed. When the participants made a seven-point confidence rating from “very sure female” to “very sure male” as a source memory measure, the results fit a model that suggests source memory as a continuous process rather than a thresholded process. Other studies have used similar procedures to show that hippocampal BOLD activation likewise best fits a continuous rather than thresholded model for source memory [50]. Nevertheless, no consensus on a wholly continuous model for source memory retrieval has been reached. Studies with similar procedures have resulted in data consistent with a threshold model for source retrieval, but also showed that, when successful, it can have varying levels of precision or accuracy [30, 51]. Continuous models for source memory retrieval are also supported by studies that show the source judgments at test can be based on either familiarity or recall in certain conditions [1, 17]. In particular, familiarity, a graded process, can be used to support memory for the source of the cues when the test is a recognition test, as in this study when there can be recognition of the correct quadrant.

Our data support graded or continuous models for source memory by showing that even when verbal report of object source has failed, source information can be revealed by study of gaze direction. It is not clear from the current data that the graded source memory retrieval processes revealed by our gaze direction data are outside the awareness of the subjects. It is entirely possible it is outside awareness, particularly considering recent data showing subjects are unable to recognize or report on their own eye movements [52]. However, there are also studies showing that people withhold responses about their explicit recollections depending on their confidence levels or other criteria [53]. Thus, eye gazes towards the correct target area when source responses are inaccurate or when participants say they have no location memory could be due to attention related to explicit partial or false source information retrieval as well as to source information processing that occurs outside conscious awareness. What is unambiguous about our data is the usefulness of eye movements in their capacity to show attentional processes during memory of source information.
